# Screening for Chlamydia and Gonorrhea in Youth Correctional Facilities, Utah, USA

**DOI:** 10.3201/eid3013.230712

**Published:** 2024-04

**Authors:** Cara Wolf, Jennifer Clifton, Xiaoming Sheng

**Affiliations:** University of Utah, Salt Lake City, Utah, USA

**Keywords:** Chlamydia, gonorrhea, Utah, youth correctional facilities, juvenile justice, adolescent medicine, sexual health, sexually transmitted infections, STIs, STDs, jails, prisons, United States, bacteria, Chlamydia trachomatis, Neisseria gonorrhoeae

## Abstract

We reviewed data obtained in October 2021–May 2023 from youth who reported a history of sexual activity upon admission to 1 of 12 juvenile justice facilities in Utah, USA, that offered screening for *Chlamydia trachomatis* and *Neisseria gonorrhoeae*. Urinalysis revealed *C. trachomatis* positivity of 10.77%, *N. gonorrhoeae* positivity of 1.08%, and coinfection *C. trachomatis N. gonorrhoeae*) of 0.90%. Prevalence of infection was similar for youths in rural and urban facilities. A total of 12.01% of those identifying as male and 14.01% of those identifying as female tested positive for *C. trachomatis,* *N. gonorrhoeae*, or coinfection. Of young adults who tested positive, 74.65% received their results while incarcerated, all of whom accepted treatment. Our research underscores the feasibility of providing prompt *C. trachomatis*/*N. gonorrhoeae* screening and treatment in juvenile correctional facilities. The pervasiveness of infection emphasizes the urgent need for early identification and treatment for *C. trachomatis* and *N. gonorrhoeae* in incarcerated youth nationwide.

In the United States, adolescents and young adults 15–24 years of age account for ≈50% of new sexually transmitted infections (STIs) annually (*1*). *Chlamydia trachomatis* is the most common bacterial STI nationwide (*2*). In Utah, USA, where our research was conducted, in 2021, two thirds of all incident *C. trachomatis* cases occurred in persons 15–24 years of age (*3*). The same year, persons 15–19 years of age experienced a higher *C. trachomatis* burden (1,399 cases/100,000 population for those identifying as female and 394 cases/100,000 population for those identifying as male) than Utah’s general population (322 cases/100,000 population overall; 426 cases/100,000 population for those identifying as female and 248 cases/100,000 population for those identifying as male) (*3*).

*Neisseria gonorrhea* is the second-most reported bacterial STI in the United States, and its incidence has increased over the past decade (*2*). Although the incidence of *N. gonorrhoeae* in Utah remains lower than the national average, *N. gonorrhoeae* rates have increased by 1,009% from 2012 to 2021 (16 cases/100,000 population in 2012 to 108 cases/100,000 population in 2021) (*3*). In 2021, the incidence of gonorrhea in Utah was relatively low among persons 15–19 years of age (152 cases/100,000 population for those identifying as female and 97 cases/100,000 population for those identifying as male) (*3*). Those rates contrast with those in other age groups in Utah, in which the male population typically has twice the incidence of gonorrhea infections compared with the female population (*3*). The Centers for Disease Control and Prevention (CDC) recommends that STI surveillance statistics within the 2020–2021 timeframe warrant careful interpretation, taking into account disruptions in STI screening and prevention during the COVID-19 pandemic (*4*).

Both *C. trachomatis* and *N. gonorrhoeae* are pathogens of public health concern because of antibiotic resistance, rising community spread, and their effects when left untreated (*5*–*7*). In female patients, untreated *C. trachomatis* and *N. gonorrhoeae* can cause pelvic inflammatory disease, which can result in infertility and chronic pelvic pain (*5*–*7*). In male patients, *C. trachomatis* and *N. gonorrhoeae* can result in epididymitis, prostatitis, and infertility (*5*–*7*). In both sexes, the sequelae of *C. trachomatis* and *N. gonorrhoeae* include reactive arthritis, proctitis, conjunctivitis, and a higher risk for acquiring more serious infections such as HIV (*7*,*8*).

A 2022 systematic review showed that adolescents in juvenile detention centers are at a higher risk for STIs than for the general population and that STI screening in correctional settings is cost-saving, is feasible, and should be performed immediately upon intake as an opt-out screening (*9*). Adolescents are at a higher risk for acquiring *C. trachomatis* and *N. gonorrhoeae* than adults for various physical, psychosocial, and behavioral reasons. The cervical mucosa in adolescent females is more susceptible to infection than in older women (*10*). Incomplete prefrontal cortex development in adolescents can lead to risky and impulsive sexual behaviors, such as unprotected sexual intercourse (*11*). In addition, adolescents may receive varied education on sexual health, which can result in a lack of knowledge regarding safe sex practices and STI prevention (*12*).

The CDC recommends that, in correctional settings, all female inmates ≤35 years of age and all male inmates <30 years of age be screened for *C. trachomatis* and *N. gonorrhoeae* upon intake (*13*). Youth in correctional facilities have a higher prevalence of both infections compared with their nonincarcerated peers, making screening vital (*13*). The National Commission on Correctional Healthcare’s 2022 Standards for Health Services in Juvenile Detention and Confinement Facilities recommends screening for STIs (chlamydia, gonorrhea, HIV, syphilis, and trichomoniasis) on arrival or within 24–48 hours of admission, basing screening decisions on local prevalence data and consultation with health departments (*14*). Our study aimed to investigate the prevalence of *C. trachomatis* and *N. gonorrhoeae* among sexually active youth in juvenile correctional facilities in Utah and the feasibility of providing timely treatment for positive cases before youth were released into the community. The University of Utah and the Department of Human Services Institutional Review Boards deemed this program exempt.

## Methods

### Setting

During October 2021–May 2023, youth who self-reported having ever engaged in sexual intercourse were offered urine screening for *C. trachomatis* and *N. gonorrhoeae* during the routine medical intake process at 12 youth correctional facilities in Utah. Before that period of data collection, *C. trachomatis* and *N. gonorrhoeae* screening had been in place since 2015, with different protocols (*15*).

According to Utah Code 26B-7-214, minors can consent to medical care for the diagnosis and treatment of STIs without a parent or legal guardian’s consent; however, a minor must believe themselves to be afflicted by an STI to consent to screening (*16*). Thus, we offered screening to minors who self-reported ever having been sexually active. The 12 correctional facilities from which data were collected were classified by the state as rural (n = 4) or urban (n = 8) (*17*). The nurse and nurse practitioner project leads helped each facility order supplies, including urine testing kits (Aptima Inc, https://www.aptima.com), prefilled laboratory forms, and mailing supplies. Facilities also were provided with a detailed manual that included stepwise instructions for the nurses involved with this provision of care. State appropriations supported this screening program.

### Exclusion Criteria

The only exclusion criterion was youth reporting never engaging in sexual intercourse. Youth under the influence of drugs or alcohol were excluded from the intake *C. trachomatis* and *N. gonorrhoeae* screening because of their inability to consent; they were later offered screening if they were still present in the facility, not intoxicated, and not undergoing withdrawal symptoms.

### Screening Process

A routine medical intake process in Utah’s juvenile correctional facilities, which involves a nurse asking youth about their health history, current illnesses, and medications, also involves screening for STIs within 24 hours of admission. In the context of our research, this nurse-led process occurred after the new inmate was admitted into the facility’s living quarters and occurred in the privacy of the medical office. Nurses inquired about history of sexual activity and assault and then offered *C. trachomatis* and *N. gonorrhoeae* urine screening for all youth who reported a history of sexual intercourse.

Nurses verbally explained the following:

1. Chlamydia and gonorrhea are common infections in persons in adolescents and young adults. Those infections often show no symptoms, leaving many persons unaware of infection. The medical team recommends screening for all persons who have ever had sexual intercourse.
2. Urine testing for chlamydia and gonorrhea is free of charge, and medical personnel keep results confidential. The urine test is not a drug test.
3. If a person tests positive for infection, they receive oral or injectable antibiotics at the facility. If a person is released before receiving their test results, the facility’s health department will contact them to arrange treatment if test results are positive for infection (nurses obtained a phone number on a written consent form).

After written consent was obtained, new inmates were given a specimen cup to provide a urine sample in a restroom near the medical office. Nurses sent specimens to a single state laboratory, and results were known within 48–72 hours in urban facilities and within 6 days in rural facilities. The extended turnaround times in rural areas were a result of the increased distance for specimen transport to the state laboratory. Youth who screened positive were either treated by the nursing staff on-site or, if they had been released, were referred to their local health department for follow-up. Nurses used standing orders to treat positive test results within the facility.

## Data Analysis

We analyzed data obtained from October 2021–May 2023. We used χ^2^ or Fisher exact tests to assess the associations and statistical significance between categorical variables. We set statistical significance at p<0.05.

## Results

We reviewed results from 557 collected urine samples. The average age of the participants who provided urine samples was 15.87 years. Of those, 69.13% were male and 28.34% were female; the remaining population identified as transgender or other gender identities. The combined positivity rate for *C. trachomatis* and *N. gonorrhoeae* was 12.75% (Table 1). Positivity for *C. trachomatis* was 10.77%, for *N. gonorrhoeae* was 1.08%, and for coinfection was 0.90%. There were no significant differences in *C. trachomatis* and *N. gonorrhoeae* infection positivity between the 8 urban (12.19%) and 4 rural (13.85%) facilities (p = 0.5762) (Table 2).

**Table 1:** Results of testing for chlamydia and gonorrhea among newly incarcerated youth, Utah Juvenile Justice & Youth Services, Utah, USA, October 2021–May 2023*

Test results	No. (%)
Negative	486 (87.25)
Positive for chlamydia	60 (10.77)
Positive for gonorrhea	6 (1.08)
Positive for chlamydia and gonorrhea	5 (0.90)

*Total sample size = 557.

**Table 2:** Combined frequency chlamydia and gonorrhea infection among newly incarcerated youth in rural versus urban locations, Utah Juvenile Justice & Youth Services, Utah, USA, October 2021–May 2023*

Test results	County classification, no. (%) patients†	
	Rural	Urban
Negative for chlamydia and gonorrhea	168 (86.15)	317 (87.81)
Positive for chlamydia or gonorrhea	27 (13.85)	44 (12.19)
Total	195 (100.00)	361 (100.00)

*Total sample size = 556; p = 0.5762 by χ^2^ test. †Missing location data from 1 case.

### Treatment for Positive Results

A total of 74.65% of youth testing positive were treated in correctional facilities, and 25.35% were released before the results were known. There were no treatment refusals within the correctional facilities, and all youth testing positive were treated by nursing staff within 2 days of receiving results. We found no significant difference in the completion of on-site treatment between urban and rural locations, despite differences in turnaround time (Table 3). Data on postincarceration treatment by the Utah Health Department were not available; thus, the proportion of positive results treated after release is unknown.

**Table 3:** Chlamydia and gonorrhea treatment among newly incarcerated youth in rural versus urban locations, Utah Juvenile Justice & Youth Services, Utah, USA, October 2021–May 2023*

Treatment	County classification, no. (%) patients†	
	Rural	Urban
Treatment provided	19 (70.37)	34 (77.27)
No treatment provided	8 (29.63)	10 (22.73)
Total	27 (100.00)	44 (100.00)

*Total sample size = 71; p = 0.5163 by χ^2^ test. †Missing location data from 1 case

### Gender Identity and STI Positivity

We found no significant difference in STI prevalence (*C. trachomatis,* *N. gonorrhoeae*, or coinfection) between female and male participants (p = 0.5242). Female inmates experienced slightly higher positivity (14.01%) than did male inmates (12.01%). Conclusions cannot be drawn for gender-diverse persons because of insufficient sample sizes (Table 4).

**Table 4:** Chlamydia and gonorrhea positivity among newly incarcerated youth by self-reported gender identity, Utah Juvenile Justice & Youth Services, Utah, USA, October 2021–May 2023*

Test results	Gender identity, no. (%) patients				
	Male	Female	Transgender	Nonbinary	Other
Negative for chlamydia and gonorrhea	337 (87.99)	135 (85.99)	4 (100.00)	2 (100.00)	7 (87.50)
Positive for chlamydia or gonorrhea	46 (12.01)	22 (14.01)	0	0	1 (12.50)

*Self-report of gender identity was optional. Total sample size = 554.

## Discussion

Our findings substantiate previous findings that incarcerated adolescents have a high prevalence of STIs (*11*,*13*). The high infection burden underscores the urgent need for early detection and treatment, particularly considering the vulnerability of incarcerated youth. This research also demonstrates the feasibility of providing prompt STI screening and treatment in carceral settings and reveals that youth were amenable to receiving this treatment. Expeditious treatment has the potential to reduce the community burden of STIs and prevent health sequelae of untreated infections.

Justice-involved youth often have various physical and mental health needs because of the lack of care received in their communities (*18*). Ideally, the time spent in incarceration could help address acute, chronic, and preventative care needs, including sexual health. Although sexual health resources exist in communities, many youth underutilize them for such reasons as embarrassment, anonymity concerns, transportation, and cost. Moral concerns among communities in Utah regarding the dissemination of sexual health education materials to adolescents is another challenge affecting the availability of such materials for this population. The state’s abstinence-only education policy is a potential contributor to this gap. To fill those educational and care gaps, healthcare providers must be comfortable discussing sexual health with adolescents and young adults.

Barriers to the implementation of this screening program in 2015 included the initial reluctance of correctional administrators to support screening for and treating asymptomatic infections, low sexual health literacy and distrust among youth, and the need to train nurses on age-appropriate interview skills (*15*). Although support for such programs is expanding among correctional administrators, nurses continue to face challenges in counseling youth with low sexual health literacy. Nurses participating in our screening efforts received education from various sources, including a Title X Family Planning Program clinic educator, on providing information to youth in an age-appropriate and trauma-informed manner. Training developed by the US Department of Health and Human Services’ Title X program, a federal program dedicated solely to family planning and related preventive health services, includes an overview of STIs, common symptoms and complications of untreated infections, STI treatment, STI prevention, and strategies for counseling youth about sexual health.

A notable limitation of this program is that we could not offer universal *C. trachomatis* and *N. gonorrhoeae* screening to all minors because of state law preventing minors from consenting to STI screening and treatment unless they suspect that they are infected, potentially hindering early STI detection and prevention. To address those constraints, nurses questioned youth about their sexual activity and recommended *C. trachomatis* and *N. gonorrhoeae* screening for persons who have ever been sexually active. This approach could have introduced a response bias, because young persons may have felt shame or embarrassment, leading to underreporting of sexual activity. In addition, the possibility of asymptomatic infection may go beyond the cognitive abilities of minors. Such limitations potentially result in missed screenings. Advocating for changes to current laws in Utah that would both protect minors’ rights and ensure access to essential healthcare services, including universal STI screening, could help address this limitation.

Our program excluded extragenital (pharyngeal and rectal) *C. trachomatis* and *N. gonorrhoeae* screening at the time of intake. For the general US population, the CDC recommends screening for *N. gonorrhoeae* pharyngeal infections and for *C. trachomatis* and *N. gonorrhoeae* rectal infections in men who have sex with men and consideration of extragenital screening for females <25 years of age; however, no guidelines exist for extragenital testing specific to the context of correctional facilities (*19*). To enhance the detection and timely treatment for extragenital infections, future iterations of the screening program we implemented at these facilities will prioritize expanding intake screening based on behavior and sites of exposure. Within 2 weeks of admission, a routine physical examination was conducted by each facility’s lead nurse practitioner in the medical office’s examination room. After gathering a detailed sexual history and identifying pertinent sexual health risks, the nurse practitioners selectively ordered extragenital *C. trachomatis* and *N. gonorrhoeae* screening, along with screening for other STIs, such as trichomoniasis, HIV, syphilis, hepatitis C, and hepatitis B.

The initial decision to perform urine testing over testing vaginal swab specimens among female youth was based on patient convenience and not wanting to contribute to sexual trauma in this population. Vaginal swab specimens offer higher sensitivity compared with urine screening in detecting both *C. trachomatis* and *N. gonorrhoeae*, with sensitivities reaching 94.1% (*C. trachomatis*) and 96.5% (*N. gonorrhoeae*) for vaginal swab specimens and 86.9% (*C. trachomatis*) and 90.7% (*N. gonorrhoeae*) for urine specimens. (*20*). Program modifications are underway to offer the option of vaginal screening for *C. trachomatis* and *N. gonorrhoeae* to female youth entering Utah correctional facilities, as well as to offer intake screening for trichomoniasis to female youth and screening for HIV in both sexes.

A final limitation was the unknown treatment completion for youth who were released before receiving positive results. Efforts are underway to improve communication with community providers and the health department regarding treating young persons testing positive after release; this system will aid in identifying barriers to sexual healthcare within the community.

## Conclusions

This research demonstrates that identifying and promptly treating *C. trachomatis* and *N. gonorrhoeae* infections in youth confinement settings is feasible. The high prevalence of infection in this vulnerable population emphasizes the importance of screening programs, timely treatment for young persons testing positive, and expansion of sexual health education for young persons both in and outside of correctional facilities. Future endeavors should focus on tracking incarcerated youth who test positive after release and identifying barriers to community-based treatment. Collaboration between correctional administrators and healthcare providers is pivotal in expanding and sustaining programs aimed at improving the well-being of justice-involved youth.
